# *Streptomyces*: implications and interactions in plant growth promotion

**DOI:** 10.1007/s00253-018-09577-y

**Published:** 2018-12-29

**Authors:** Oluwaseyi Samuel Olanrewaju, Olubukola Oluranti Babalola

**Affiliations:** Food Security and Safety Niche Area, Faculty of Natural and Agriculture Sciences, North-West University, Mmabatho, 2735 South Africa

**Keywords:** *Actinomycetes*, Biocontrol, Biofertilizer, *Streptomyces*, Sustainable agriculture, VOCs

## Abstract

With the impending increase of the world population by 2050, more activities have been directed toward the improvement of crop yield and a safe environment. The need for chemical-free agricultural practices is becoming eminent due to the effects of these chemicals on the environment and human health. Actinomycetes constitute a significant percentage of the soil microbial community. The *Streptomyces* genus, which is the most abundant and arguably the most important actinomycetes, is a good source of bioactive compounds, antibiotics, and extracellular enzymes. These genera have shown over time great potential in improving the future of agriculture. This review highlights and buttresses the agricultural importance of *Streptomyces* through its biocontrol and plant growth-promoting activities. These activities are highlighted and discussed in this review. Some biocontrol products from this genus are already being marketed while work is still ongoing on this productive genus. Compared to more focus on its biocontrol ability, less work has been done on it as a biofertilizer until recently. This genus is as efficient as a biofertilizer as it is as a biocontrol.

## Introduction

Actinomycetes are Gram-positive bacteria characterized by a genome with high G + C ratio. They are mostly aerobic, but some of them can grow anaerobically. Several actinomycetes form branching filaments and possess mycelial growth while some species produce external spores. Out of all rhizosphere microbes, actinomycetes are regarded to be special in plant growth promotion because they exhibit many useful traits (El-Tarabily and Alkhajeh 2016; Monteiro et al. 2017). Their filaments and ability to sporulate help them cleave strongly to the rhizospheric soil particles forming a strong bond with the plants.

Actinomycetes are a numerous and widely distributed group of soil microbes, constituting to about 10 to 50% of the soil microflora community. Tyc et al. (2017), Adegboye and Babalola (2012), and other researchers have reported them to be important producers of secondary metabolites. The metabolites produced are diversified in their biological activities and functions such as antifungal, insecticidal, antibacterial, and antihelminthic activities. Actinomycetes, like other plant growth-promoting microbes, also produce phytohormone (Jog et al. 2016), and solubilize phosphate (El-Tarabily et al. 2008; El-Tarabily and Sivasithamparam 2006). This goes a long way to show the interest in the use of actinomycetes that can solubilize phosphate in phosphate-deficient soils. As reported by Mercado-Blanco and Bakker (2007), they interact with plants as free-living non-symbiotic bacteria. Despite the fact that they are largely spread in nature and have been strongly studied largely due to their production of numerous antibacterial and antifungal compounds, only a few works have established their importance, for example, wheat (Toumatia et al. 2016) and broccoli (Ruzzi and Aroca 2015). Due to their abundance, they have been isolated from tissues of various plants including wheat, rice, maize, soybean, and pepper (Adegboye and Babalola 2012; El-Tarabily and Alkhajeh 2016; Goudjal et al. 2014; Jog et al. 2016).

Most studied actinomycetes plant growth-promoting species possess antibacterial or antifungal activity which was imminent during their screening as biocontrol agents (Adegboye and Babalola 2012; Jog et al. 2016). To buttress this, many products such as Mycostop (*Streptomyces griseoviridis* K61), Actinovate (*Streptomyces lydicus*), and Nogall (*Agrobacterium radiobacter* Strain K1026) have been produced. Actinomycetes’ ability to colonize plant roots, fight against pathogens, synthesize extracellular proteins, produce antibiotics, and degrade phytotoxins makes them potent plant growth-promoting agents.

## Properties and classifications of actinomycetes

They include microbes delineating an expanse range of morphologies, from hyphal forms to coccoid including those exhibiting highly variable physiological and metabolic traits. Actinomycetes members have evolved lifestyles differing to that of pathogens as demonstrated by *Corynebacterium*, *Mycobacterium*, *Nocardia*, *Tropheryma*, and *Propionibacterium*. They are soil inhabitants (*Streptomyces*) and gastrointestinal commensals (*Bifidobacterium*) as well as plant commensals (*Leifsonia*) (Ventura et al. 2007).

These bacteria resemble fungi in their morphology forming branching hyphae, asexual spores, and mycelium. This means that they have characteristics that are common to both fungi and bacteria; they are actually at the transition between bacteria and fungi. They are abundant and widely distributed in the soil leading to the claim that the characteristic smell of soil is actually due to the actinomycetes present. Some form mutualistic relationships with plants promoting their growth and protecting them from pathogens. In addition, they also form associations with green algae. However, not all actinomycetes are beneficial microbes, some are plant pathogens causing diseases such as potato scab, wilt, and gall, as well as causing diseases in humans and animals. They are classified into different genera based on their physiology and morphology. Based on this, some of the known actinomycetes are *Actinomyces*, *Nocardia*, *Streptomyces*, *Thermoactinomyces*, *Waksmania*, *Thermopolyspora*, *Micromonospora*, *Thermomonospora*, *Actinoplanes*, and *Streptosporangium* (Babalola et al. 2009). Among the actinomycetes, the *Streptomyces* genus happens to be the most regarded and well known due to its numerous identified importance. Its importance has been established in health, agriculture, and other important sectors. In this review, emphasis will be on this genus in relation to its plant growth promotion abilities as a biocontrol agent and biofertilizer. We will also consider the strategies common to their actions as plant growth promoters.

## Overview of the *Streptomyces* genus

*Streptomyces* are important groups of soil bacteria from the actinomycetes family. Alongside *Micromonospora*, they are the most commonly described actinomycetes making up 1–20% of the culturable soil microbes. The colony growth of *Streptomyces* becomes visible when a spore germinates and produces long filaments which have multiple nuclei (van Dissel et al. 2014). The filaments elongate by apical growth and branch repeatedly producing a substrate mycelium that develops both on the culture medium and into it. The hyphae, which are formed by the mycelium, penetrate the medium in the presence of extracellular hydrolytic enzymes solubilizing organic molecules present. The filaments allow for efficient use of nutrients in the rhizosphere and it also enables the *Streptomyces* to colonize substrates better than unicellular microorganisms. Cell interactions are important in governing multicellular development and cellular differentiation in the *Streptomyces* colony (Miguélez et al. 2010). At the latter stage when the colony is getting old and the nutrients are exhausted, specialized branches evolve from the surface of the colony which results in the aerial mycelium (reproductive) that grows vertically into the air (Hu et al. 2012). Advances in sequencing technology have revealed significant findings which have helped in understanding the mechanisms involved at different transition stages of *Streptomyces* life cycle although the focus has mainly been on single-species cultures. On the other hand, same growth level cannot be recorded in the laboratory for any individual soil microbe because the laboratory environment may not capture the full potential of these microbes due to the difference in environmental conditions and the satiated level of microbes in the soil.

An extremely important and notable characteristic of *Streptomyces* is the ability to carry out a complex life cycle that can phylogenetically be considered as one of the probably several pioneer attempts at multicellular transformations and evolvement (Jones and Elliot 2017). They act in the catabolism of complex molecules and substances like lignocellulose, xylan, cellulose, and lignin, which are important in soil organic matter catabolism.

*Streptomyces* is one of the major sources of bioactives known and they are majorly studied for this reason (Tom et al. 2016). Some of the bioactives include secondary metabolite production, in the form of antibiotics and extracellular enzymes not forgetting antitumor and agroactive compounds which are important in the decomposition of cellulose and chitin (Adegboye and Babalola 2012; Tyc et al. 2017).

Approximately two thirds of natural antibiotics have been isolated from actinomycetes, and about 75% of them are from the *Streptomyces* genus (Franco-Correa et al. 2010). It is also reported by Berdy (2005) that *Streptomyces* produce about 7600 bioactive compounds. This has made *Streptomyces* become the major antibiotic producer used for drug discovery and production in the pharmaceutical industries (Kumari et al. 2017; Shekh and Naim 2017). Most *Streptomyces* produce some minute amount of α-butyrolactones, which is analogous to homoserine lactones, and have been deduced to play an important role as signals and markers for the setting-in of morphological and physiological differentiation (Safari et al. 2014). α-Butyrolactone, which is present in most *Streptomyces*, was the first *Streptomyces* sporulation factor discovered. It was first characterized as an activator of streptomycin production and spore formation in *Streptomyces griseus* (Miguélez et al. 2010).

## Plant growth-promoting *Streptomyces* (PGPS)

Most *Streptomyces* are efficient rhizosphere and rhizoplane colonizers. They can also be endophytes colonizing inner tissues of host plants (Sousa and Olivares 2016). These attributes may be due to features such as quorum sensing controlled gene expression, multiplication rate, antibiotics, siderophore, cellulases, phytohormones, amino acid synthesis, chitinase, lipase, and β-1,3-glucanase production. Exudate attraction of *Streptomyces* to the rhizosphere is accomplished by the chemotaxis movement of these microbes.

In the agricultural sector, the emergence of PGPS either as biofertilizer or biocontrol has led to new discoveries into other ways these microbes can be useful. *Streptomyces* are not left out in this discovery, although many studies have focused on the biocontrol activities of these genera due to its high production of bioactive compounds which are used as defense mechanisms.

### PGPS as a biocontrol agent

The global attempts to discovering natural products as biocontrol agents for plant protection have notably been on the rise and actinomycetes, *Streptomyces* being the most proactive, appear to be a readily available natural choice in finding new ways to combat plant pathogens.

Their abilities to control plant pathogens stem from the following traits:

- Synthesis of plant growth regulators (Goudjal et al. 2013)
- Siderophore production (Vijayabharathi et al. 2015)
- Antibiotics production (Couillerot et al. 2013)
- Volatile compound secretion (Jones and Elliot 2017) and
- Competition for nutrients

Its main biocontrol ability is attributed to its strong production of antibiotics, volatile compounds, and other metabolites which help in its role as antipathogens, e.g., siderophores from *S. coelicolor* (Som et al. 2017), chitinase from *S. violaceusniger* YH27A strain (Gherbawy et al. 2012), as well as the antifungal nigericin, and antibiotic geldanamycin from *S. violaceusniger* YCED-9 (Shrivastava and Kumar 2018). These compounds are attributed to hypha development activated by a nutrient deficit. Their biological activities are well described in the works of Al-Askar et al. (2015), Errakhi et al. (2016), and Shekh and Naim (2017). In like manner, they are also regarded as stress metabolites because of their role in adaptivity during stress. Production of these metabolites involves the actions of some genes that exist majorly in clusters. It is in these clusters that regulatory proteins and phosphorylated guanosine nucleotide phosphate (ppGpp) (which is an important protein for metabolite synthesis) are encoded (Sivapragasam et al. 2017). Nutritional stress activates alarmone ppGpp which regulates antibiotic production. *Streptomyces* antibiotic regulatory proteins and lysosomal acid lipase families, which are species specific, are also important in metabolism pathway regulations. The type of signals sent and received by a *Streptomyces* determines the metabolites produced. Nutrient deficit sends a signal, pathogen attack also activates a signal, etc. In PGPS cell–cell communication, α-butyrolactones are the major signaling molecules. Some *Streptomyces* exhibiting biocontrol activities against some known plant pathogens are shown in Table 1.

**Table 1:** Biocontrol and plant growth-promoting activities of some PGPS

	PGPS	Elicited effects	References
Biocontrol activities	*Streptomyces griseus*	*Rhizoctonia solani*	Merriman et al. (1974)
	*Streptomyces kasugaensis*	*Fusarium* sp.	de Vasconcellos and Cardoso (2009)
	*Streptomyces* J-2		Errakhi et al. (2016)
	*Streptomyces* sp.	*Sclerotium rolfsii*	Gholami et al. (2014)
	*Streptomyces sanglieri*	*Ganoderma boninense*	Azura et al. (2016)
	*Streptomyces griseorubens* E44G	*Fusarium oxysporum* f. sp. *lycopersici*	Al-Askar et al. (2015)
	*Streptomyces rochei* ACTA1551		Kanini et al. (2013)
	*Streptomyces felleus* YJ1	*Sclerotinia sclerotiorum*	Cheng et al. (2014)
Plant growth-promoting activities	*Streptomyces anulatus* S37 *Streptomyces* sp.	Grapevine Bean Chickpea	Couillerot et al. (2013)
			Jarak et al. (2012)
			Gopalakrishnan et al. (2015)
	*Streptomyces matansis* BG5, *Streptomyces* sp. RSF17, *Streptomyces vinaceus* CRF2, *Streptomyces* sp. CRF14, *Streptomyces pulcher* CRF17, *Streptomyces griseoincarnatus* SCF18		Javaid and Sultan (2013)
	*Streptomyces* PRIO41	Pepper	Robles-Hernández et al. (2015)
	*Streptomyces mutabilis*	Wheat	Toumatia et al. (2016)
	*Streptomyces fumanus* gn-2	Soybean	Doolotkeldieva et al. (2015)

Pathogen-antagonistic PGPS were also used to promote the growth of coniferous plants. In Brazil, there was the report of an isolate close to *Streptomyces kasugaensis* which was shown to inhibit the growth of *Fusarium* and *Armillaria* pine rot and it also showed plant growth promotion abilities on *Pinus taeda* seedlings under greenhouse experiment (de Vasconcellos and Cardoso 2009). Studies of El-Abyad et al. (1993) also showed the use of *Streptomyces pulcher*, *Streptomyces canescens*, and *Streptomyces citreofluorescens* in biocontrol of diseases caused by *Fusarium oxysporum*, *Verticillium albo atrum*, *Alternaria solani*, *Pseudomonas solanacearum*, and *Clavibacter michiganensis* subsp. *michiganensis* in tomatoes. It was reported that tomato growth was significantly improved. Another example was observed evident in the study conducted by Tokala et al. (2002) with the *Streptomyces lydicus* strain WYEC 108 in both growth chamber and greenhouse experiments. The result of this study was later used in the formulation and the commercialization of Actinovate® and Actino-Iron®, a well-known biocontrol product because of its unique traits (Crawford et al. 2005). It was observed that there was an increase in shoot and root length, and root wet weights in pea seedlings. There was also an increase in root nodulation, nodule size, and number of *Rhizobium* spp. As this was feasible in the more numerous and vigorous nodules found in PGPS-colonized plants than in the control plant, an increase in the number of bacteria per nodule, nitrogenase activity, and nodular assimilation of iron was also observed (Tokala et al. 2002). This is also supported by the work of Hoster et al. (2005). This ability is due to the production of antibiotics and enzymes (such as the chitinolytic enzymes) to inhibit pathogens, and of plant growth-promoting compounds like phytohormones, solubilization of phosphates, and competition with plant pathogens for substrates and nutrients (Charousová et al. 2016; de Vasconcellos and Cardoso 2009; Kinkel et al. 2012).

PGPS has also been widely used in the biocontrol of soil-borne fungal pathogens (Gopalakrishnan et al. 2013). The biocontrol ability against numerous phytopathogens *Fusarium oxysporum*, *Penicillium digitatum*, and *Sclerotium rolfsii* among others is well documented (Al-Askar et al. 2015; Priya et al. 2017). Their secondary metabolite-producing ability is the most important property of this genus in carrying out their biocontrol activities.

### PGPS as biofertilizer

Despite their popularity as biocontrol agents, their functioning as biofertilizers has been partly understudied. Being one of the most abundant microbes in the microflora, and considering their effectiveness in plant root system colonization, it is surprising that they have not been adequately studied for plant growth promotion. They directly promote plant growth by the production of phytohormones (auxins, cytokinins, and gibberellins), siderophores scavenge ferric iron from the environment, nitrogen fixation and, suppression of stress in plant by production of 1-aminocyclopropane-1-carboxylate (ACC) deaminase activity (Sadeghi et al. 2012; Verma et al. 2011). The use of *Streptomyces griseus* in plant growth was reported by Merriman et al. (1974). In the work, the isolate was originally meant to serve as biocontrol but it was found out to have a tremendous effect on grain yield, dry foliage weight, tiller number, and advanced head emergence for both wheat and oat over controls especially when applied on carrots. Biocontrol efficacy of *Streptomyces* sp. against bacterial, *Fusarium* and *Verticillium* wilts in tomato was also reported by El-Abyad et al. (1993). In the study, tomato growth was significantly observed to improve due to the growth regulators produced by the inoculum. The release of phosphate through the actions of released malic acid and gluconic acids by *Streptomyces* mhcr0816 and *Streptomyces* mhce0811 respectively was reported in the study of Jog et al. (2014). Like most rhizobacteria, they are capable of directly affecting the growth of plants positively. Other works on PGPS acting as plant growth promoters on different crops are shown in Table 1.

### Specific mechanisms of PGPS which can be exploited for plant growth promotion

A new discovery was made as a result of the interaction between PGPS and various yeast species. The co-culture resulted in a previously unknown reaction in PGPS cultures. This reaction which is glucose-repressible was called exploratory growth behavior by the PGPS which occurs as a result of deficient glucose in the PGPS-yeast environment. This means that glucose deficiency activates PGPS exploration through yeast stimulation (Jones et al. 2017). A rapid outgrowth of vegetative hyphae is a characteristic of exploratory growth behavior as observed in *S. venezuelae* which was estimated to be approximately 90 mm h^−1^ (Jones et al. 2017). On a closer look, the exploring hyphae do not seem to branch which may contribute to the spreading of the exploring PGPS colonies. This outward spreading is an advantage in the organism dominating its immediate environment by taking more space, while in the rhizosphere, the colony is able to outcompete other microbes through this exploratory growth mechanism. Perhaps, this might be the reason for its abundance compared to other soil microbes.

Aside from the exploratory growth mechanism, PGPS use volatile organic compounds (VOCs) to modulate environmental conditions through their actions as signals. These signals can result in regulating gene expression of surrounding microbes. They can act as elicitors of gene activation or repression as well as determine the response of other microbes in the soil environment. Furthermore, VOCs along with trimethylamine act as antibacterial and antifungal agents. Both *Micrococcus luteus* and *Bacillus subtilis* were being inhibited by the synergy of PGPS and trimethylamine (Jones et al. 2017). Trimethylamine raises the pH of the environment to a level that is not conducive to other microbes thereby affecting the transmembrane pH and proton motive forces.

### Identification of candidate PGPS

Most *Streptomyces* species can be wrongly misjudged as good plant growth promoters based on their ability to produce metabolites. In most cases, these metabolites are virtually not important because they have not been proven to be useful in anyway. Again, not all metabolites produced are actually good for the plants, some are actually deleterious to plant health. In view of this, we briefly propose a “check” to identify important PGPS taking into consideration time factor, cost of experiments, and purpose of experiment.

One of the most important traits of a good plant growth promoter is root colonization (Bhattacharyya and Jha 2012; Olanrewaju et al. 2017). Although it has been reported that *Streptomyces* are good root colonizers, some are not and the degree also varies from one species to another. Before a species can be selected as a plant growth-promoting agent, the level of root colonization must be ascertained. It must be able to compete favorably well against other microbes to be able to exert its influence on the host plants.

We cannot underestimate the importance of the metabolites these species produce. As said earlier, not all metabolites have positive impacts. Using advanced tools and technologies, the genetic makeup and gene expression level in the organisms can be studied. Knowing this will help identify important genes, metabolites, and invariably functions of these genes. This can be made possible through the application of functional genomics and system biology. Cost and time have to be taken into consideration in this regard. Although advent of next generation sequencing (NGS) has substantially reduced the cost of sequencing, not all researchers can still afford to spend that amount on testing an organism. Since most *Streptomyces* genes are in clusters, these clusters can be identified including their mode of regulations. In Table 2, we can find examples of some species, their metabolites, and the gene locations.

**Table 2:** Some *Streptomyces* species, their gene clusters, metabolites, and functions of these metabolites

Species	Gene clusters	Metabolites reported	Functions of metabolites	References
*S. griseus*	Streptomycin biosynthetic genes	Streptomycin	Antibacterial	Lee et al. (2018)
	Grixazone biosynthetic genes	Grixazone B	Produced as a parasiticide during low availability of phosphate	Arakawa (2018), Tsujimoto et al. (2016)
		A factor	Induces the production of other secondary metabolites	Fiebig et al. (2018), Takano et al. (2016)
*S. rochei*	Lankacidin biosynthetic genes	Lankacidin C	Antibacterial	Lu et al. (2018)
	Lankamycin biosynthetic genes	Lankamycin	Antibacterial Antitumor	Lu et al. (2018)
*S. avermitilis*	Avermectin polyketide synthase	Avermectins	Antimicrobial Antiparasitic Pesticide	Cheng et al. (2018), Choi et al. (2018), Rath et al. (2018), Zhang et al. (2015)
*S. clavuligelus*	herABCDEFG genes Cephamycin biosynthetic genes pcbAB	Clavulanic acid	Antibacterial	Flores-Gallegos and Nava-Reyna (2019), Romero-Rodríguez et al. (2018)
		Cephamycin C		
*S. coelicolor*	abeABCD; α-abeAgenes	Actinorhodin	Antibacterial	Čihák et al. (2017), Gao et al. (2012), Huang et al. (2001), Moore et al. (2012)
	Prodiginines biosynthetic genes	Prodiginines	Antimalarial	
	Albaflavenone biosynthetic genes	Albaflavenone	Antibacterial	
	SCO7221	Germicidin A		
*S. venezuelae*	Chloramphenicol biosynthetic genes Jadomycin biosynthetic genes	Chloramphenicol Jadomycin B	Antibacterial	Cytryn et al. (2017), Mousa and Raizada (2015), Thanapipatsiri et al. (2016)
*S. cinnamonensi*	Monensin biosynthetic genes	Monensin	Growth-promoting agent Herbicide	AlMatar et al. (2017), Zhang et al. (2016)
*S. rimosus*	Oxytetracycline biosynthetic genes	Tetracyclines	Antibacterial	Lee et al. (2015), Petković et al. (2017)
*S. aureofaciens*		Aureomycin	Antibacterial	Francis (2017)
*S. lydicus*		Selamectin	Parasiticide Antihelminthic	Selvakumar et al. (2014)
*S. hygroscopicus*	Geldanamycin biosynthetic genes	Geldanamycin	Antitumor	He et al. (2008)

Molecular markers can also be useful in this case. Regions coding for important traits that are valuable in the *Streptomyces* can easily be mapped and studied. Various molecular markers have been developed such as restriction fragment length polymorphism (RFLP), small nucleotide polymorphism (SNP), and diversity array technology (DArT) markers.

Application of NGS technology with molecular markers can be used in the identification of gene clusters present in *Streptomyces*. Once clusters can be correctly identified then the genes present in these clusters will definitely be known. Once known, functions of each gene can be known through functional genomics, proteomics, transcriptomics, and other omics technologies. Interactomics can be applied to know the interactions between the genes and subsequent responses to these interactions. The studies of these interactions can open up the pathway for the synthesis of the major bioactive compounds produced by each species in response to various interactions.

## PGPS: antibiotic resistance, safety, and mode of application

Antibiotics are used to control invading microbes in humans, plants, and animals. Majority of these antibiotics are gotten from the *Streptomyces* genus. In the soil, these genera are relatively dominant. These antibiotics when secreted by soil *Streptomyces* help ward off invading pathogens. Not all antibiotics released are actually safe for the plants they are meant to protect. Some are actually deleterious to the plants. Effects of antibiotics on the environment are not yet well ascertained. On plants, toxicity of some antibiotics has been assayed and various negative and positive impacts have been recorded both on the plants and the soil. Variations were also observed in the activities of some antibiotics toward various plants as seen in the plant growth promotion of tetracycline on radish yields and the same antibiotics decreasing pinto yield (Batchelder 1982). The current interest of cutting down on chemical fertilizer use has increased the use of plant growth-promoting rhizobacteria (PGPR) as alternatives.

Development of antibiotic resistance in various sectors such as health, agriculture, and environment has led to some researchers suggesting the possibility of pathogens becoming resistant to PGPS. Since the basic mode of action of these PGPS against pathogens is the production of antibiotics and they are the largest producers of antibiotics, any development of resistance to these strains will have great impact on health and agricultural sectors. Pathogens can adapt to the antibiotics after long and frequent interactions by developing resistance. According to Martínez (2008), the release of a large amount of antibiotics might change the rhizosphere population dynamics as well as selection of resistance. This notably implies that concentration of the antibiotics in contact with the pathogens in the rhizosphere is key to the development of resistance. In as much as no concrete evidence have evolved concerning development of antibiotic resistance toward PGPS, there can be transfer of genes (horizontal and lateral) among various individuals in the rhizosphere. With this in mind, there is the possibility of these pathogens picking up resistance genes and thereby eliciting resistance against the particular antibiotics. This is so very possible but it is not enough to suggest that the pathogen will therefore be able to resist the PGPS because PGPS do not release only one type of antibiotics. If the pathogen develops resistance to a particular antibiotic, there are many more that will act against the pathogen either from the same PGPS or other PGPS present in the rhizosphere. In addition to antibiotics, PGPS and other biocontrol agents produce enzymes and various volatile compounds that are used as mechanisms of biocontrol.

Due to some of the metabolites produced, some safety concerns have been raised. Safety to the plants and humans to exposure to PGPR interactions is becoming a concern in certain quarters. No health issues either to plants or humans have been reported as a result of the use of any PGPR. Although various metabolites are produced as biocontrol agents, none of these have been associated with any negative effect on plants. Most metabolites causing diseases are from pathogens. An example is seen in pyocyanin which is produced by *Pseudomonas aeruginosa*, an opportunistic plant and human pathogen (Schroth et al. 2018; Stover et al. 2000). They are also regarded as plant growth promoters but they have been reported to induce growth of some plants (Tiwari and Singh 2017).

Most experiments on PGPR focus on the use of the microbes other than their products. This is better because it is these microbes that release the metabolites that are used as biocontrol agents or as growth promoters. As part of their mechanisms of actions, they produce antibiotics, volatile compounds, auxins, gibberellins, cytokinins, ethylene, enzymes, etc. (Olanrewaju et al. 2017), which are used in plant growth promotion. Some research have focused on using some of these antibiotics directly on plants against pathogens and for plant growth promotion (Devireddy et al. 2017; Minden et al. 2017). The use of these antibiotics directly on soil can increase the rise of resistance in the soil (Cadena et al. 2018; Zhang et al. 2017). It is safer to use the PGPRs instead of their products as no negative effect have been recorded from their use. Application of the PGPR directly will also mean that all other mechanisms in the PGPR will be available for the plant use than selected metabolites which will act singly.

## Conclusion and recommendations

Using plant growth-promoting bacteria to improve nutrient availability, biosynthesize metal chelators and phosphorus solubilizers, produce phytohormones, control phytopathogens, and alleviate abiotic stress in plants is an important practice for sustainable agriculture and also a good alternative to environmentally hazardous chemical fertilizers and pesticides (Olanrewaju et al. 2017). Except for few cases, PGPR are safe to the plants and humans.

The importance of *Streptomyces* strains in plant growth promotion is vividly expressed in their antipathogenic activities. Their metabolic potential should be a strong area to be looked at by researchers as it is paramount to majority of the plant growth promotion traits that have been discussed in this review. It is of little surprise that this rich source of bioactive compound-producing microbes has been underutilized so to say in agriculture. We believe that these microbes should be considered a great weapon in the search for viable plant growth promoters for sustainable agriculture.

Aside from trimethylamine, other VOCs produced by *Streptomyces* should be ascertained for their ability to alter environment pH as this might be another area to explore in biological control. More *Streptomyces* strains can be cultured and exploited toward their VOC production. VOCs represent a largely untapped class of metabolites, and further work will be required to fully appreciate the ecological roles of these compounds and agricultural potential.

Exploration by *Streptomyces* can also be targeted as it allows for a rapid and vast area covering by the organism which enhances closeness to the nutrient source. Communication has also been established by the production of certain VOCs. As discussed earlier, exploration results from interaction through the glucose-deficient phenomenon which means that different interactions when studied might pave way for new developments to be discovered which might be helpful in plant–microbe and microbe–microbe interactions for effective plant growth promotion.

The overall adaptive traits of the *Streptomyces* genus which make it possible to exist in a wide range of both aquatic and terrestrial environments can be attributed to the formation of spores. This trait gives it a greater edge over other microorganisms. The ecophysiological significance of the interactions between the genus and various parts of plants which confers its plant growth promotion traits should be well studied to vividly elucidate the mechanism involved.

Most of the activities of the *Streptomyces* genus come from its ability to produce secondary metabolites; therefore, mechanisms involved in the regulation and production of these metabolites as well as the regulation of the gene clusters should be well studied with the advent of next-generation sequencing and advancement in bioinformatics. Omics approaches will definitely contribute to the existing knowledge on *Streptomyces* rhizosphere colonization, crosstalk between the genus, and other organisms in the rhizosphere, etc.

Finally, research into bioinoculants formulation involving carriers, additives, and optimum delivery methods will increase the chances of these organisms to survive when introduced into the environment thereby increasing their acceptability.

There is no precise means to differentiate plant growth-promoting microbes as some microbes are termed as plant growth promoters and pathogens. Example of such is *Pseudomonas aeruginosa* that has been reported as both plant growth promoter and pathogen (Tiwari and Singh 2017). There should be a clarity between these two groups as they can all be found in the rhizosphere and endosphere of plants. Research should be directed toward better understanding and differentiation of these groups.

Although no report has emerged of pathogens being resistant to any biocontrol agent, control measures should be put in place as a check.
